# Supplementary material for: Lung Function Testing and Prediction Equations in Adult Population from Maputo, Mozambique
Source: Int J Environ Res Public Health. 2020 Jun 24;17(12):4535. doi: 10.3390/ijerph17124535 (PMC7344554; doi:10.3390/ijerph17124535)
Supplement: Supplementary file 1 [file ijerph-17-04535-s001.zip › ijerph-767205-SI.docx]

Supplementary Materials:


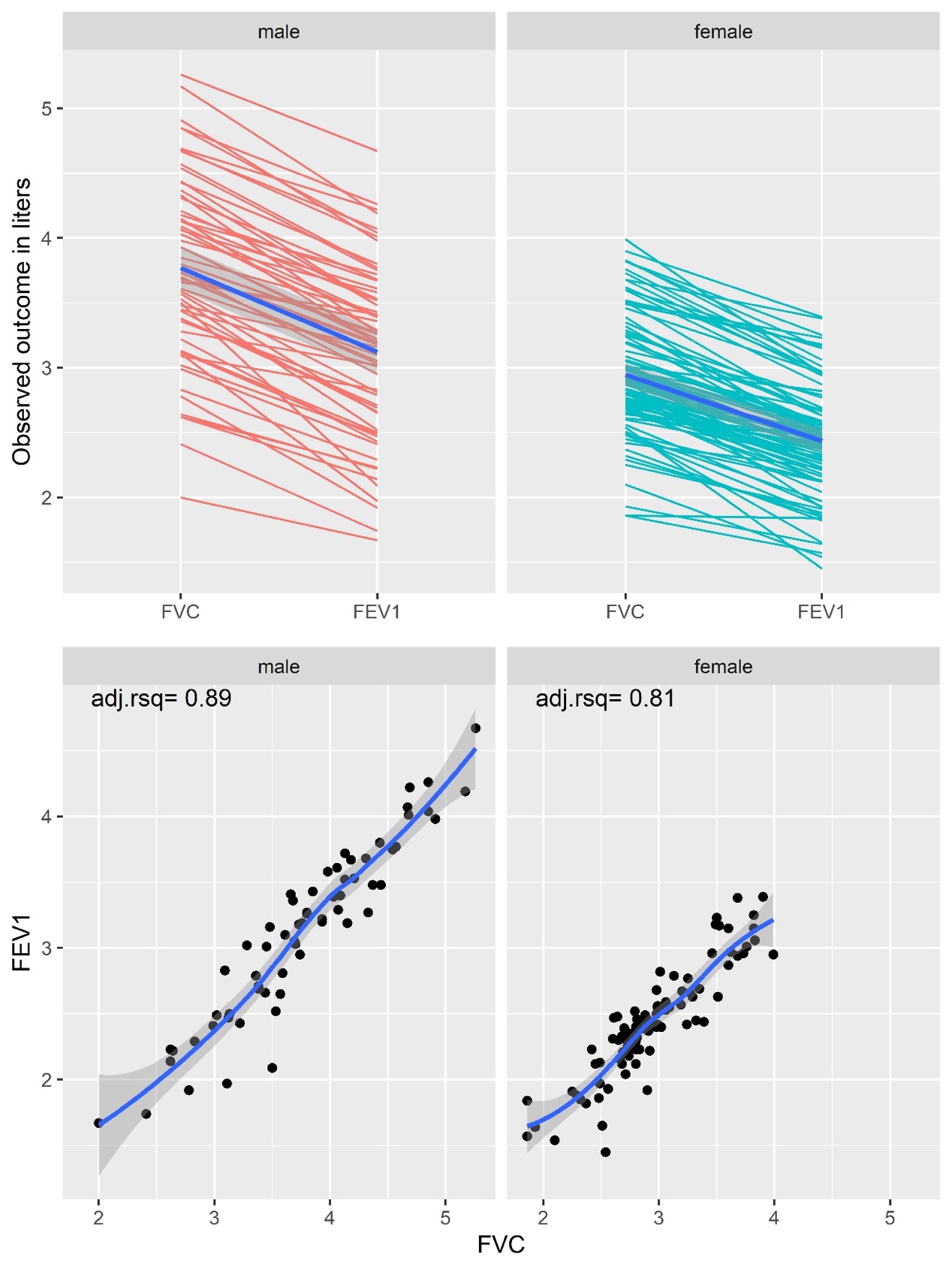


**Figure S1.** Association between the observed spirometry outcomes (FVC and FEV1) according to sex.

Legend: There is a strong association between the values for FVC and FEV1 in both sexes.


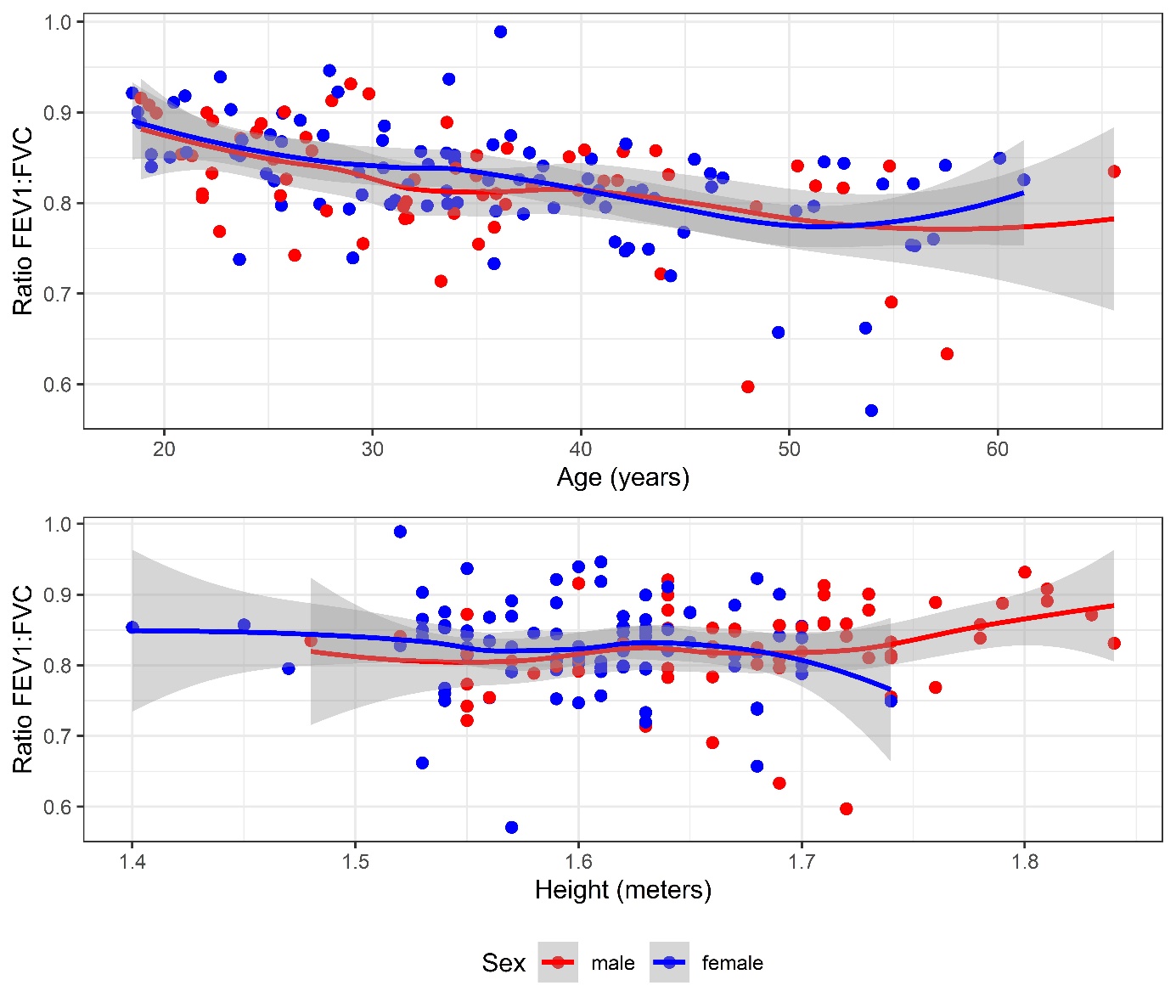


**Figure S2.** Relationship between the ratio of FEV1/FVC with age and height.

Legend: A decreasing trend (LOESS fit) with age (negative slope) is observed for the FEV1/FVC ratio; however, no particular trend with height is visible (almost flat line). No difference across sex is visible in both trends.


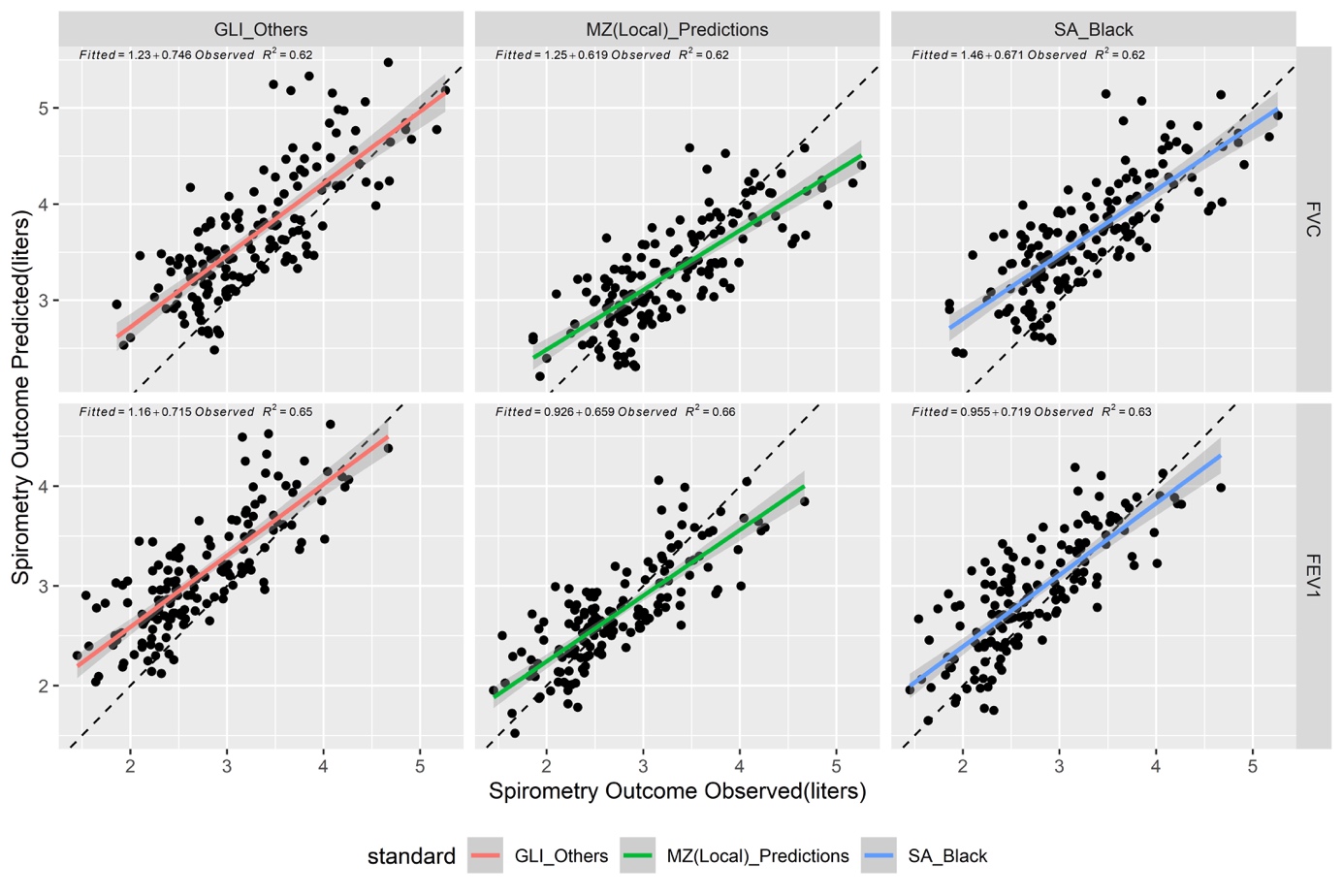


**Figure S3.** Goodness of fit for Mozambican (Local) standards predictions and that based on GLI or South African standards, when compared to the observed data.

Legend: This figure shows the performance of the standards in terms of fitting to the observed data. We depict the diagonal line (Fitted= 0+ 1* Observed), which would indicate values where observed is exactly equal to the fitted. The coloured lines represent the regression equation for Fitted based on the Observed data. For the South African Black and the GLI Others standards, we observe more points above the diagonal line indicating that the predicted values are higher than the observed values. This is more balanced for the Mozambican Local standard based predictions. In addition, we also demonstrate through this plot that the predictions based on Mozambican standards are lower than the South African Black and the GLI Others on average.


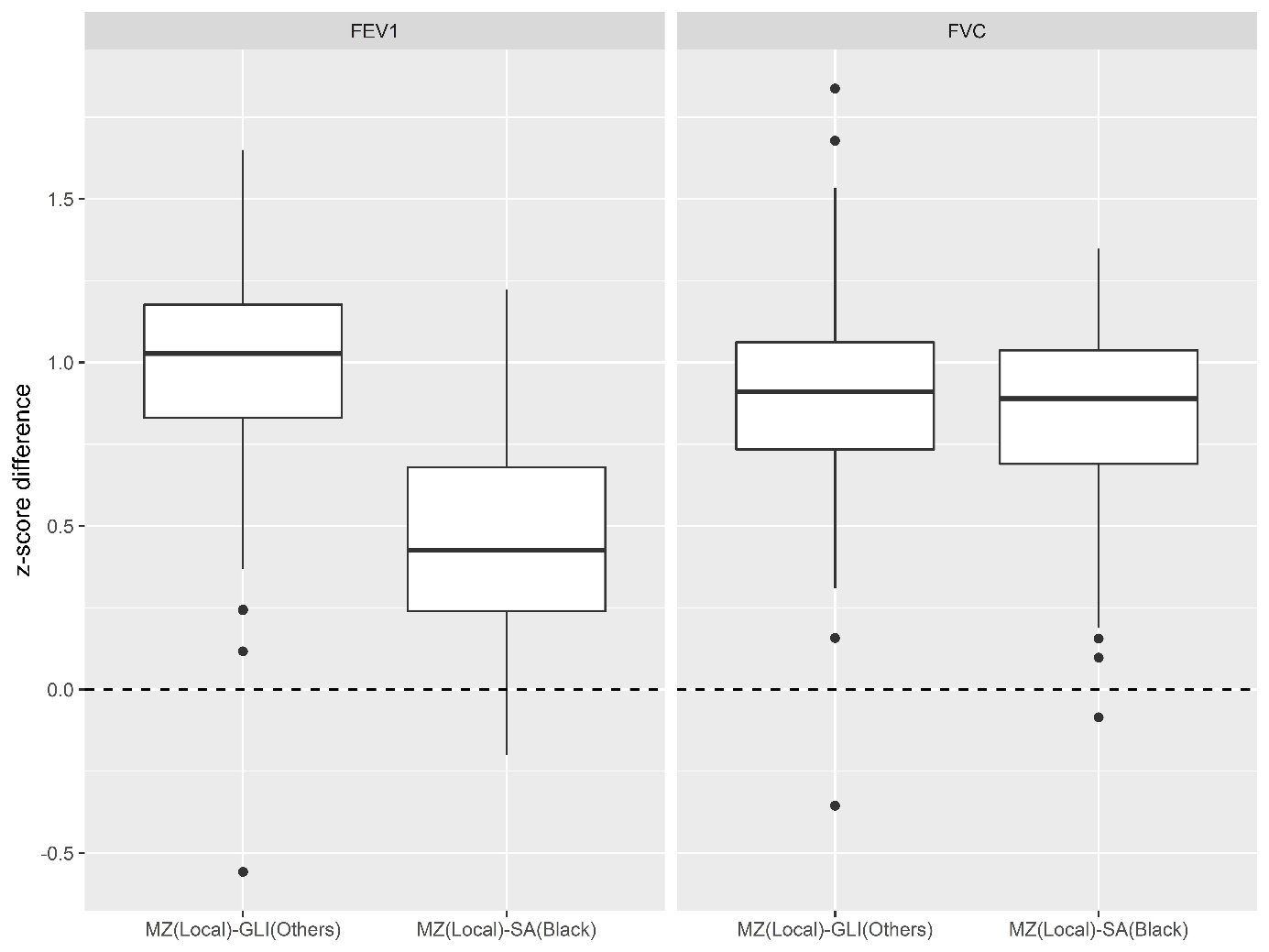


**Figure S4.** Differences between z-scores of Mozambican standards and z-scores based on GLI or South African standards.

Legend: The box plots show the distribution of the differences in z-scores for FEV1 and FVC based on the Mozambican prediction equation compared to GLI and South African equations, respectively. On average, the difference between Mozambican predictions and the other predictions is about one standard deviation (z-score difference=0.9) for FVC. For FEV1 the difference with GLI prediction is 1.2 standard deviations and only about 0.4 standard deviation compared to South African predictions.


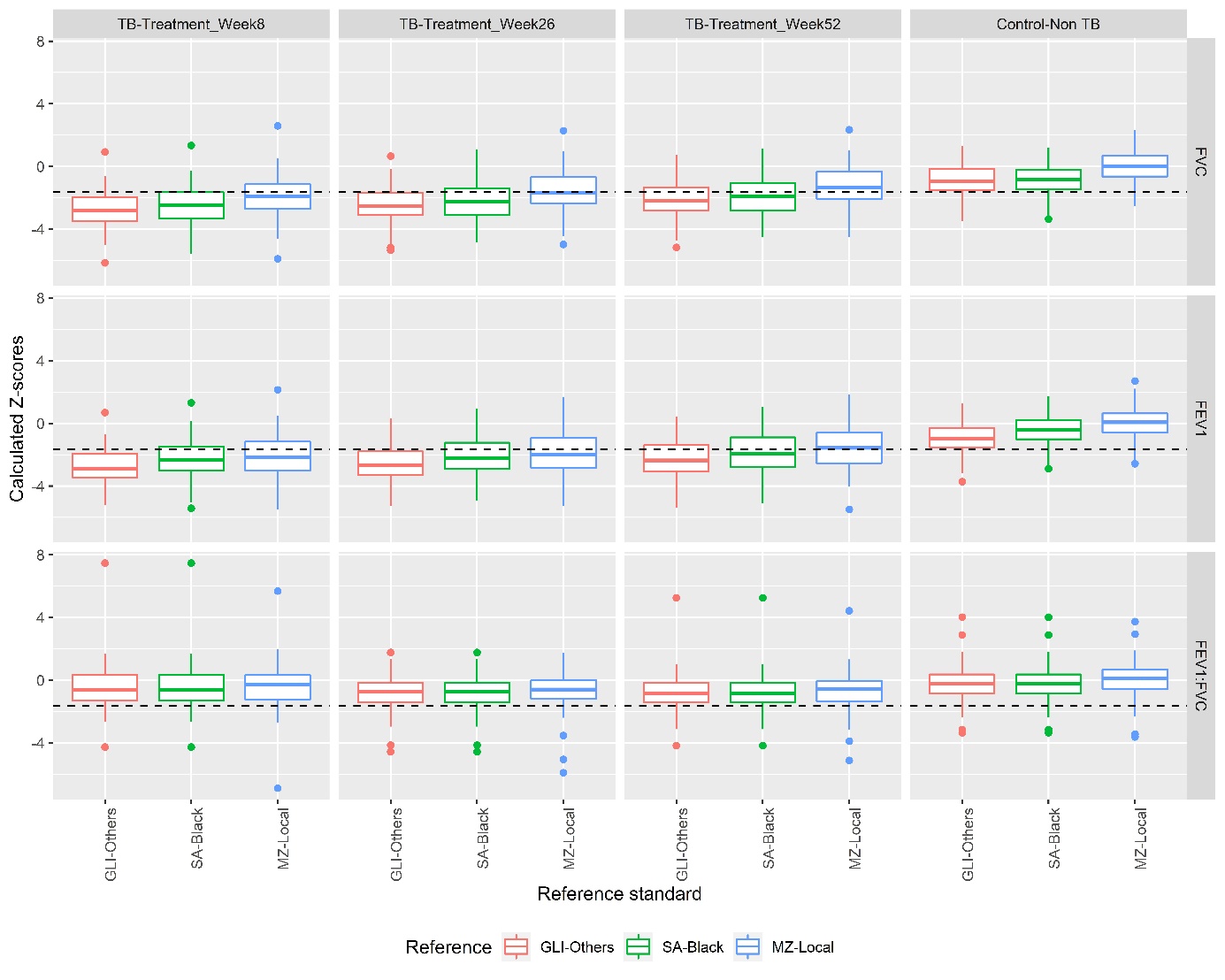


**Figure S5.** Comparison of performance of local Mozambican, GLI and South African standards on a data set of a TB cohort during and after TB treatment from Maputo, Mozambique.

Legend: The plot of the z-scores on the TB cohort (Week 8, Week 26 and Week 52 from TB treatment start) shows an improvement for FEV1 and FVC over time, as we would expect. The difference across the standards used (GLI - Others, South African-Black and local Mozambique) seen in the TB cohort is similar to the control group, which constitutes the healthy volunteer local population in Mozambique. The effect on the FEV1/FVC ratio does not differ across the different standards in both the TB cohort and in the control group, which is in accordance with the GLI guidelines (Quanjeer et al. 2012), where they describe the lack of ethnicity effect.

**Table S1.** Likelihood Ratio Test Results for selecting the regression model covariates.


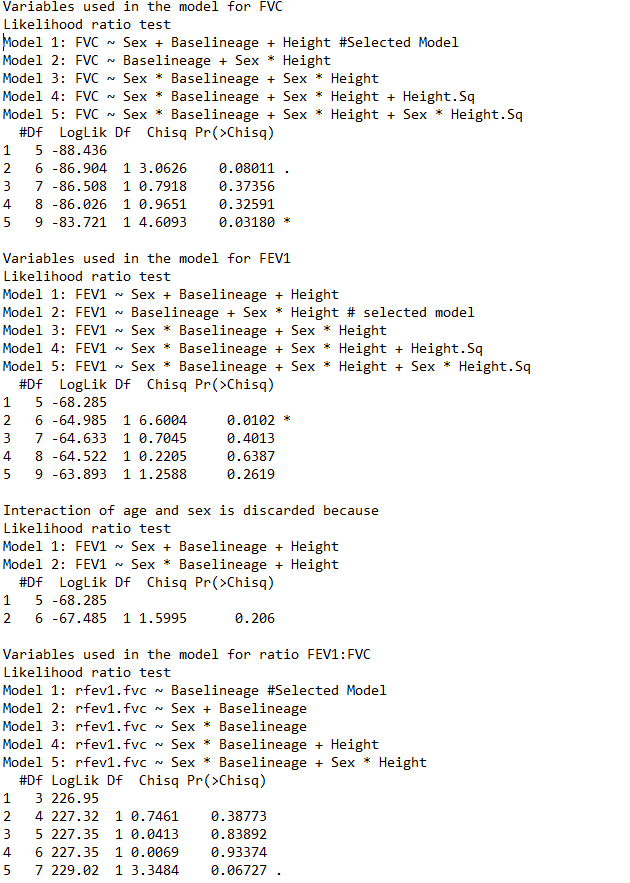


**Table S2.** Absolute numbers of participants with different spirometry outcomes and severity grades depending on the applied reference standard (Mozambican, GLI - *Others* or South African Black prediction equations).

| ***Mozambique (Local)*** | ***GLI-Others*** | | | | ***South Africa-Black*** | | | |
| --- | --- | --- | --- | --- | --- | --- | --- | --- |
| ***Impairment type*** | Normal | Obstruction | Restriction | Mixed | Normal | Obstruction | Restriction | Mixed |
| Normal (139) | 113 | 1 | 23 | 2 | 116 | 2 | 20 | 1 |
| Obstruction (9) | 0 | 8 | 0 | 1 | 0 | 8 | 0 | 1 |
| Restriction (7) | 0 | 0 | 6 | 1 | 0 | 0 | 6 | 1 |
| ***Impairment severity*** | Normal | Mild | Moderate | Severe | Normal | Mild | Moderate | Severe |
| Normal (139) | 113 | 24 | 1 | 1 | 116 | 22 | 1 | 0 |
| Mild (15) | 0 | 4 | 8 | 3 | 0 | 7 | 7 | 1 |
| Moderate (1) | 0 | 0 | 0 | 1 | 0 | 0 | 0 | 1 |

Legend: Compared to the local Mozambican standard (139), a smaller number of participants was classified as having normal lung function if GLI (113) or the South African (116) prediction estimates were used as reference on the Mozambican sample. Also, compared to the local Mozambican standard (1), the number of participants with moderate and severe lung impairment was increased when GLI (14) and South African (10) references were applied.

**Table S3.** Sensitivity analysis of the prediction equations based on the observed Mozambican data.

| **Outcome** | **Training 2/3;**  **Testing 1/3 –**  **1 Random Draw** | **Leave One Out Cross**  **Validation - LOOCV** | **10-Fold**  **Cross-Validation** | **5 Repeated 10-Fold**  **Cross-Validation** |
| --- | --- | --- | --- | --- |
| FVC | Rsquared-0.63;  RMSE-0.37;  MAE-0.30 | Rsquared-0.60;  RMSE-0.44;  MAE-0.35 | Rsquared-0.62;  RMSE-0.43;  MAE-0.35 | Rsquared-0.64;  RMSE-0.43;  MAE-0.35 |
| FEV1 | Rsquared-0.68;  RMSE-0.33;  MAE-0.27 | Rsquared-0.67;  RMSE-0.37;  MAE-0.30 | Rsquared-0.67;  RMSE-0.37;  MAE-0.30 | Rsquared-0.66;  RMSE-0.37;  MAE-0.30 |
| Ratio FEV1/FVC | Rsquared-0.26;  RMSE-0.05;  MAE-0.04 | Rsquared-0.20;  RMSE-0.06;  MAE-0.04 | Rsquared-0.28;  RMSE-0.06;  MAE-0.04 | Rsquared-0.27;  RMSE-0.06;  MAE-0.04 |

Legend: Sensitivity analysis of the prediction equations based on the observed Mozambican data under different cross-validation methods for evaluating the predictive performance of the models. The predictions performances are consistent across different methods. These outcomes can be compared to the prediction outcomes based on 1-South African equations produce (Rsquared = 0.62; RMSE = 0.58; MAE = 0.47) for FVC, (Rsquared = 0.63; RMSE = 0.43; MAE = 0.34) for FEV1; 2-GLI equations produce (Rsquared = 0.62; RMSE = 0.60; MAE = 0.48) for FVC, (Rsquared = 0.65; RMSE = 0.54; MAE = 0.44) for FEV1 and (Rsquared = 0.21; RMSE = 0.06; MAE = 0.04) for the ratio of FEV1/FVC. The prediction efficacy is better than the South African and GLI for the outcomes of FEV1 and FVC. However, the prediction efficacy for the ratio FEV1/FVC is similar to that based on GLI.

**Table S4.** Summary from 10000 non-parametric bootstraps sensitivity analyses on the healthy adult’s data from Mozambique.

| **Summary Measure** | **Characteristics** | **FVC (95%CI)** | **FEV1 (95%CI)** | **Ratio FEV1/FVC (95%CI)** |
| --- | --- | --- | --- | --- |
| Rsquare | Model Fit | 0.62  (0.52, 0.69) | 0.66  (0.57, 0.73) | 0.22  (0.12,0.34) |
| Intercept | Coefficient | −2.27  (−4.01, −0.69) | −3.50  (−5.18, −1.45) | 0.92  (0.89, 0.96) |
| Sex Female | Coefficient | -0.49  (−0.65, −0.34) | 3.33  (0.77, 5.92) | - |
| Age | Coefficient | −0.02  (−0.03, −0.01) | −0.02  (−0.03, −0.02) | −0.003  (−0.004, −0.002) |
| Height | Coefficient | 3.99  (3.03, 5.02) | 4.43  (3.21, 5.79) | - |
| Sex Female: Height | Coefficient | - | −2.28  (−3.87, −0.72) | - |

Legend: The obtained estimates are similar to those derived from the fitted regression equation to describe the observed data. The obtained confidence intervals are overlapping with the estimates reported in Table 2 for Mozambique.

**Table S5.** Comparison of lung impairment prevalence in the own Mozambican TB cohort using Mozambican, GLI and South African prediction equations.

|  | **TB-Treatment Week 8 (N=62)** | **TB-Treatment Week 26 (N=62)** | **TB-Treatment Week 52 (N=62)** |
| --- | --- | --- | --- |
| **Impairment**  **Mozambique**  **(Local standard)** |  |  |  |
| - Impaired | 39 (66.1%) | 33 (54.1%) | 28 (45.2%) |
| - Not impaired | 20 (33.9%) | 28 (45.9%) | 34 (54.8%) |
| - Missing | 3 | 1 | 0 |
| **Impairment**  **South Africa**  **(Black standard)** |  |  |  |
| - Impaired | 46 (78.0%) | 42 (68.9%) | 40 (64.5%) |
| - Not impaired | 13 (22.0%) | 19 (31.1%) | 22 (35.5%) |
| - Missing | 3 | 1 | 0 |
| **Impairment**  **GLI (Others standard)** |  |  |  |
| - Impaired | 51 (86.4%) | 46 (75.4%) | 40 (64.5%) |
| - Not impaired | 8 (13.6%) | 15 (24.6%) | 22 (35.5%) |
| - Missing | 3 | 1 | 0 |

Legend: This table describes a lung function in a TB cohort (Week 8, Week 26 and Week 52 from TB treatment start) using different prediction equations. As expected, for all reference standards, the improvement of lung function under treatment is observed, with best lung function results at 52 weeks after TB diagnosis and treatment start. However, less lung impairment is described by the local Mozambican standard compared to South African Black and GLI - Others equations.
